# An Efficient Approach to Large-Scale Ab Initio Conformational Energy Profiles of Small Molecules

**DOI:** 10.3390/molecules27238567

**Published:** 2022-12-05

**Authors:** Yanxing Wang, Brandon Duane Walker, Chengwen Liu, Pengyu Ren

**Affiliations:** Department of Biomedical Engineering, The University of Texas at Austin, Austin, TX 78712, USA

**Keywords:** conformational energy profile, computational efficiency, semi-empirical method, neural network potential, AMOEBA force field

## Abstract

Accurate conformational energetics of molecules are of great significance to understand maby chemical properties. They are also fundamental for high-quality parameterization of force fields. Traditionally, accurate conformational profiles are obtained with density functional theory (DFT) methods. However, obtaining a reliable energy profile can be time-consuming when the molecular sizes are relatively large or when there are many molecules of interest. Furthermore, incorporation of data-driven deep learning methods into force field development has great requirements for high-quality geometry and energy data. To this end, we compared several possible alternatives to the traditional DFT methods for conformational scans, including the semi-empirical method GFN2-xTB and the neural network potential ANI-2x. It was found that a sequential protocol of geometry optimization with the semi-empirical method and single-point energy calculation with high-level DFT methods can provide satisfactory conformational energy profiles hundreds of times faster in terms of optimization.

## 1. Introduction

Conformational energy was first developed to describe the ring strain energy of hydrocarbon rings, and has been generalized to include energy coming from bond distortions, angle strain, torsional strain, etc., enabling it to describe overall deviations of molecular geometry from the ideal [1,2]. If limited to systems of biological interest, e.g., ligand-receptor binding, the conformational energy is mostly attributed to the torsional strain from bond rotations. Attaining accurate conformational energetics of molecules is of great significance to calculate and understand many important molecular properties, such as molecular dipole moment, binding affinity, etc. [3,4]. Traditionally, quantum mechanical (QM) methods are applied to obtain the conformational energy landscape of a molecule, but expensive computational cost makes direct usage only suitable for small systems.

Molecular mechanics (MM) can be applied to large biologically interesting systems by using classical potential energy functions instead of solving the Schrödinger equation [5,6,7]. The core of MM methods is utilizing a set of equations and parameters, also known as force fields (FFs), to model molecular interactions and potential energy surfaces. Typically, the parameters of FFs are derived by fitting to experimental and/or QM data, based on predefined rules of transferability [8,9,10,11,12,13]. This process needs a medium to large amount of QM calculations to obtain the conformational energetics of small model compounds. Nevertheless, some parameters, e.g., torsion parameters, are sensitive to the local chemical environment and, hence, are not as transferable [14,15]. As a consequence, to attain simulations of quality, researchers have to parameterize novel molecules individually with ab initio QM data, which is time-consuming, but can be made more efficient with automation tools [16,17,18,19], such as Poltype [18,19] for the atomic multipole optimized energetics for biomolecular simulation (AMOEBA) force field [20], the force field builder in the commercial Schrödinger software suite [21] for the optimized potentials for liquid simulations (OPLS) force field [22,23], and so on. With so much QM data to compute, researchers tend to use “poor” transferability rules for torsion to allow less QM. Thus, it becomes valuable to find more efficient alternatives to increase the scalability of computing QM for conformational energy surfaces.

As the data-driven deep learning methods are being incorporated more with FF development and molecular simulations [24,25,26,27,28,29,30], there appears to be a huge demand for giant ab initio QM datasets for training [24,31,32,33,34]. Neural network potentials (NNPs) could learn the conformational energy landscape accurately, but require vast amounts of data to achieve the desired performance. To deal with expensive dataset generation, for example, the ANI potential utilizes active-learning to intelligently let the model choose what data it wants for improvements. The dataset contains millions of DFT energy points that have been reduced, and ANI potential’s performance was further refined [25,35]. The difficulty of generating such a large dataset impedes the evaluation and application of deep learning models. Researchers are unable to generate their own data set to tailor deep learning models based on their individual needs, such as specific levels of accuracy of QM energy, hybrid models of NNP and FF, etc.

To the best of our knowledge, there are few published articles on the comparison of alternatives to speed up conformational scans without significant loss of accuracy. There has been some work on using NNPs to accelerate conformational scans and refine the torsion parameters for the GAFF2 FF [27]. However, one might not want to use the data generated by another NNP to train their own NNPs. Furthermore, we anticipated that semi-empirical methods [36,37] could be another alternative with accuracy in between DFT and MM methods. Thereby, in this work, we benchmarked the quality of the geometry and energy obtained with AMOEBA, an FF method, GFN2-xTB, a semi-empirical method, ANI-2x, an NNP method, and ωB97XD [38,39], a DFT method in ab initio conformational energy scan settings with a fragment dataset compiled by us, with both good drug-likeness and chemistry coverage considered.

## 2. Results and Discussion

### 2.1. Our Dataset Has a Broad Coverage of Chemical Space

**Figure 1:**
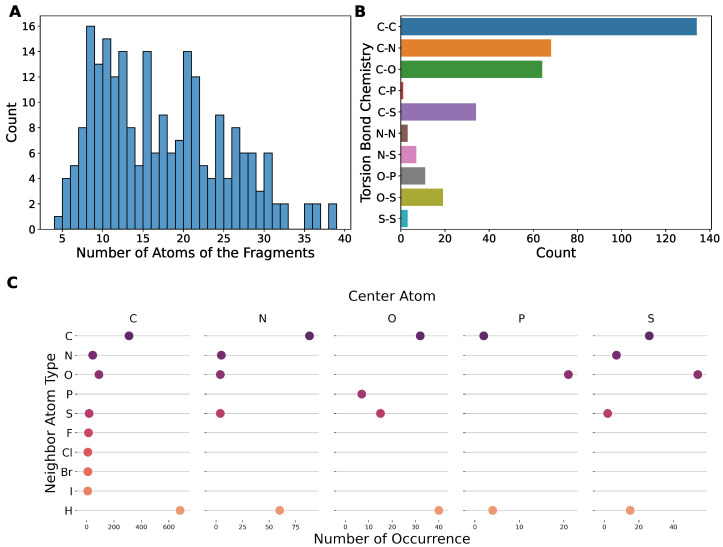
The torsion chemistry coverage of the dataset compiled in this work. (**A**) The histogram of the number of atoms of the fragments in the dataset. (**B**) The chemistry coverage measured by the elements of center atoms. (**C**) The chemistry coverage measured by the elements of neighbor atoms. The categories without a point mean that no such data exists in the dataset.

**Figure 2:**
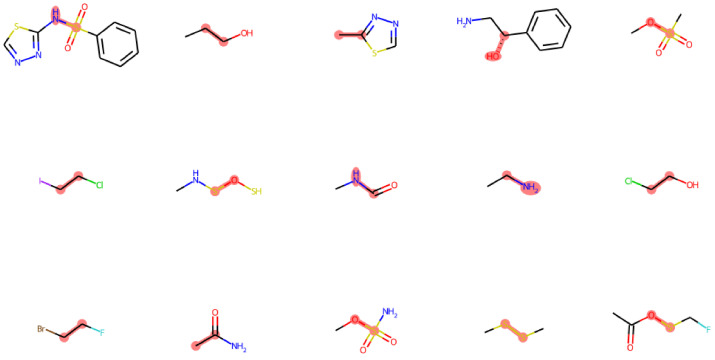
Typical fragments randomly selected from the dataset. The torsion bond and the center atoms are highlighted in red.

The dataset contains a total of 233 fragments with a broad range of sizes (Figure 1A), with the number of total atoms varying from ∼5 to ∼40 covering chemical environments of different functional groups. We also analyzed the composing elements of the center atoms (i.e., atoms *b,c* in torsion *a-b-c-d*) and the neighbors (i.e., atoms *a,d*). Here, we only considered the elements of biochemical interests, namely C, N, O, P, S, F, Cl, Br, I, and H, of which only the first five are not monovalent; therefore, only they can serve as center atoms in a torsion bond. Our dataset covers the majority of the chemistry defined by this rule (Figure 1B), except those that are unstable or that are rarely found in biology-related compounds, e.g., P-P, P-S, N-P, O-O, N-O, etc. The N-O bond is actually very common, such as in nitro groups, but it appears as terminal bond that cannot serve as a center bond (bond *b,c* in torsion *a-b-c-d*). Our dataset actually includes nitro groups, as shown in Figure 1C. This figure shows the number of occurrences of the elements of neighbor atoms grouped by the element of the center atom, which illustrates the diversity of the local environments of the torsion bonds. Nitro groups are represented in the point under the center atom N at neighboring atom O. The vacancies with no point suggest bonding environments rarely found in biological systems that were excluded from our dataset, such as hetero atoms neighbored by halogens, etc. The structures of typical fragments randomly sampled from our dataset are presented in Figure 2.

### 2.2. Cheaper Optimization Methods Can Also Provide Acceptable Geometry

**Figure 3:**
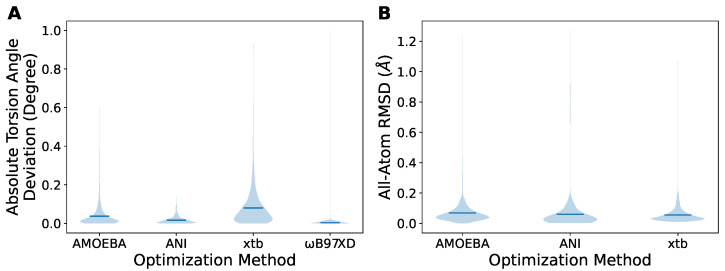
The analysis of the geometry obtained from the constrained optimizations. (**A**) The deviations of the torsion dihedral angles from the target set in the constraints. (**B**) The RMSD of the geometry obtained from the indicated optimization methods compared to the ωB97XD-optimized one. The bars in the violins represent the mean values.

We then benchmarked the three methods on this dataset with the scenario of conformational energy scans, assuming that the DFT results are the ground truth. First, we assessed the geometry obtained from the constrained optimization with AMOEBA, ANI, xtb, and ωB97XD/6-311G. Unlike DFT methods that remove the corresponding degrees of freedom for the constraints, MM methods usually restrain the degrees of freedom by adding extra harmonic potentials. Therefore, the constrained torsion dihedrals remain almost the same as the targets after ωB97XD optimization, but could change slightly after the AMOEBA, ANI, and xtb optimizations (Figure 3A). The reason is that the energy increase due to the unsatisfied restraints could be compensated for by the other interactions in the molecule. However, acceptable results where the overall deviation is within 1 degree can be achieved by setting sufficient force constants that control the steepness of the added potential wells on the constrained dihedrals. The optimized geometries were subsequently compared with the ωB97XD-optimized one (Figure 3B) by all-atom RMSD. The majority of structures showed an RMSD of less than 0.2 Å, with a few extreme RMSDs of up to ∼1 Å. In particular, xtb yielded the best agreement with ωB97XD results, suggested by the lowest mean RMSD (the bar inside the violin), although the torsion angle deviation was not as good as other methods. Generally speaking, all of the three MM methods seemed able to provide acceptable geometry for conformational scans.

Additionally, it should be noted that ANI-2x can only be applied to molecules containing C, H, O, N, S, F, and Cl elements, leading to its incapability of handling many small molecules, e.g., fragments with P, Br, and I in our dataset. We also found that ANI often fails the optimization due to convergence issues. Perhaps the reason for this is due to the optimization engine—atomic simulation environment (ASE)—that ANI employed. This further hinders researchers from utilizing ANI for generating accurate QM datasets. In contrast, the xTB method is capable of handling molecules across the periodic table with up to thousands of atoms [37]. Researchers need not bother with the chemistry coverage and scalability issues.

### 2.3. DFT Method Is Still Necessary to Obtain Satisfactory Torsion Energy Profile

**Figure 4:**
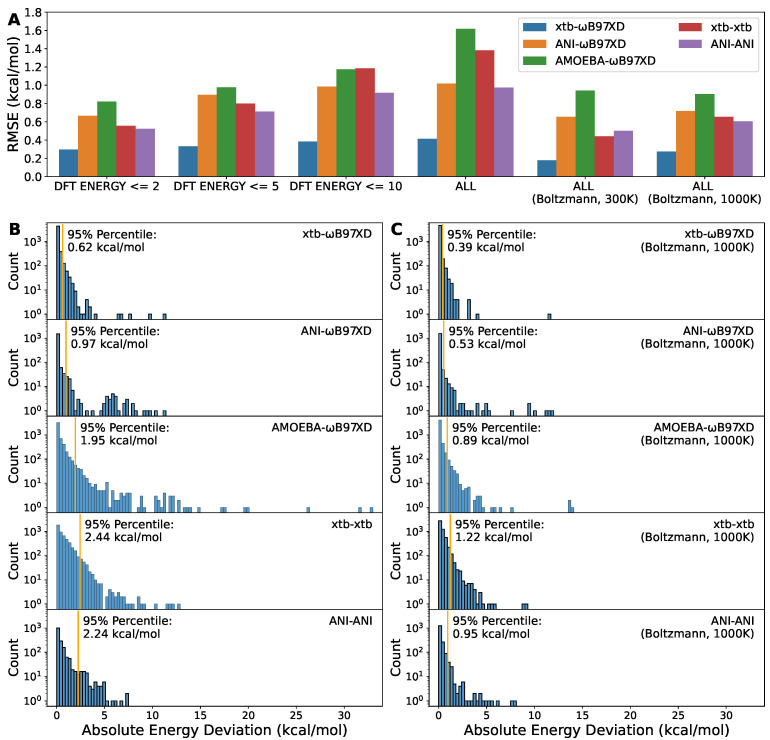
The comparison of energies obtained from various combination of optimization and single-point energy methods. (**A**) The root mean squared error of the indicated methods to DFT methods, ωB97XD-ωB97XD. This refers to ωB97XD/6-311G optimization followed by ωB97XD/6-311+G* single-point energy. The same notation convention applies to others in the legends. (**B**) The histograms of absolute errors for the indicated methods. (**C**) The histograms of Boltzmann-weighted absolute errors at 1000 K for the indicated methods. The orange vertical lines represent the 95% percentiles. The y axes were transformed to log scale to highlight outliers.

We further compared the deviation of the energies obtained through the various combinations of geometry optimization methods and single-point energy methods to the energies from DFT for both, i.e., ωB97XD/6-311G optimization followed by ωB97XD/6-311+G* single-point energy, shortened as ωB97XD-ωB97XD. This notation, *opt*-*sp*, is also used in the legend of Figure 4. As seen, the energies from xtb-xtb and ANI-ANI were in poor agreement with those from ωB97XD-ωB97XD, as suggested by the overall root mean squared error (RMSE) of ∼1 kcal/mol (Figure 4A) and the 95% percentile of >2 kcal/mol (Figure 4B). The ANI-2x paper actually reported similar numbers for the comparison between ANI-ANI and DFT-DFT [25]. The overall RMSE of ANI-ωB97XD was similar to ANI-ANI but its 95% percentile, ∼1 kcal/mol, was better than ANI-ANI, suggesting that the unsatisfactory RMSE might be attributed to the existence of the outliers (Figure 4B). Nevertheless, what excited us most is that xtb-ωB97XD demonstrates an excellent alignment to the reference, ωB97XD-ωB97XD, as indicated by the overall RMSE of 0.41 kcal/mol and the 95% percentile of 0.62 kcal/mol. The AMOEBA-ωB97XD was also included here as the baseline FF method. These results aligned well with the geometry deviation mentioned before (Figure 3), which gave us an idea of how much difference the subtle deviation in geometry could make to the torsion energy profile.

In MD simulations of ambient conditions, molecular structures fluctuate around the equilibrium conformation. Thus, it is more relevant and important to model the energy profile accurately close to the equilibrium geometry. To this end, we included RMSEs for subsets of data points with different cutoffs on DFT energies (Figure 4A). As expected, all methods performed better with decreasing of DFT energy, i.e., coming closer and closer to the equilibrium. Even for xtb-ωB97XD, the RMSE for the subset with lower DFT energy was slightly smaller than the overall RMSE. The RMSE on data points with DFT energy no greater than 2 kcal/mol is lower than 0.30 kcal/mol. In addition, we also included the Boltzmann-weighted overall RMSEs at temperatures of 300 K and 1000 K (Figure 4A) and the histograms of Boltzmann-weighted absolute deviations at 1000 K (Figure 4C). The Boltzmann-weighted RMSE and 95% percentile xtb-ωB97XD are 0.27 kcal/mol and 0.39 kcal/mol, respectively. These results were consistent with that the xTB methods were parameterized to provide good geometries [37].

### 2.4. Inexpensive Computational Cost Highlights the Advantage of the Semi-Empirical Method xtb

**Figure 5:**
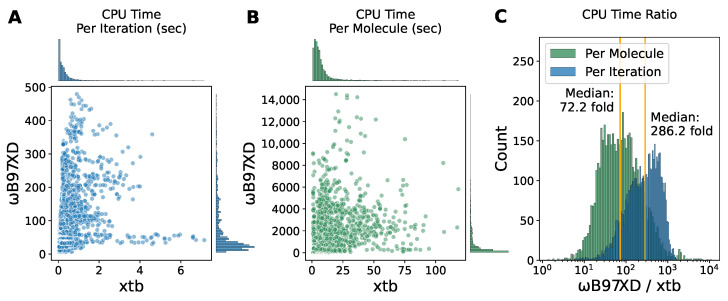
The computational cost comparison between xtb and ωB97XD optimization. (**A**,**B**) The scatter plot of the CPU times elapsed per iteration (**A**) and per molecule (**B**) for ωB97XD optimization versus xtb optimization. (**C**) The histogram of the ratio of CPU times for ωB97XD optimization to xtb optimization. The orange vertical line represents the median.

When it comes to large scale conformational energy scans, time efficiency becomes non-trivial and as important as the accuracy of energy landscapes. Based on what was discussed above, it is tempting to replace the DFT optimization with xtb but for now, the DFT method should be kept for the single-point energy calculations to ensure satisfactory energy profiles. We found that the CPU times consumed for the DFT optimization of each torsion point were mainly distributed under 100 s per iteration (Figure 5A) and under 2000 s per molecule (Figure 5B). However, for the semi-empirical method xtb, the CPU time costs were mostly less than 1 s per iteration and less than 20 s per molecule. We next calculated the ratio and found that for most of fragments in our dataset, xtb optimization is faster than DFT by a median factor of ∼72 with the majority lying within a factor of 10–1000 (Figure 5C). We also determined the elapsed times for optimization with ANI (Figure S1), which mainly lie in the range of 3.5–6.0 s per molecule. ANI was slightly faster than xtb. Since ANI does not provide an official timer, it was difficult to time each iteration.

### 2.5. Case Discussion

**Figure 6:**
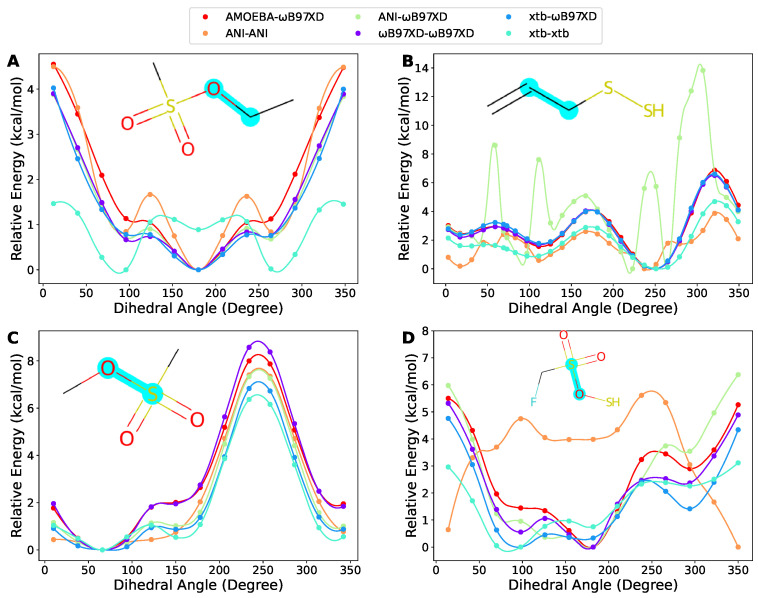
Four tricky cases containing sulfur atoms. (**A**) A fragment where ANI and xtb could provide good geometry, but not good energy. (**B**) An example where ANI was outperformed by xtb since ANI could not generate reasonable geometry. (**C**,**D**) Two examples where xtb-ωB97XD exhibited relatively bad RMSEs, but still was able to keep the overall shape of the torsion energy surfaces. The scanned torsion bonds are highlighted in cyan. The same notation as in Figure 4 is used in the legends.

Along with the overall comparison, herein, we show some of the poor cases we visually inspected. Often, the fragments containing sulfur atoms could be challenges for the methods benchmarked in this work. Figure 6A demonstrates a fragment where ANI and xtb could provide good geometry, but not good energy. Figure 6B shows an example where ANI was outperformed by xtb since ANI could not generate even a reasonable geometry, as indicated by the up and down of the ANI-ωB97XD curve, but the ANI-ANI curve suggests that ANI energy could somehow compensate for the bad geometry. In Figure 6C,D, we present two examples where xtb-ωB97XD exhibited relatively bad RMSEs (1.17 and 0.59 kcal/mol, respectively), but still was able to keep the overall shape of the torsion energy surfaces. Usually, the performance of NNPs is highly dependent on the training data. Since the dataset of ANI-2x was not publicized, we could not do much analysis. However, according to the description of the dataset creation in the ANI-2x paper, the molecules containing sulfur atoms were generated by simply substituting oxygen atoms. We assume that some functional groups containing sulfur atoms were probably not covered, such as sulfonic groups. This could be the reason why ANI-2x behaved poorly on these molecules.

**Figure 7:**
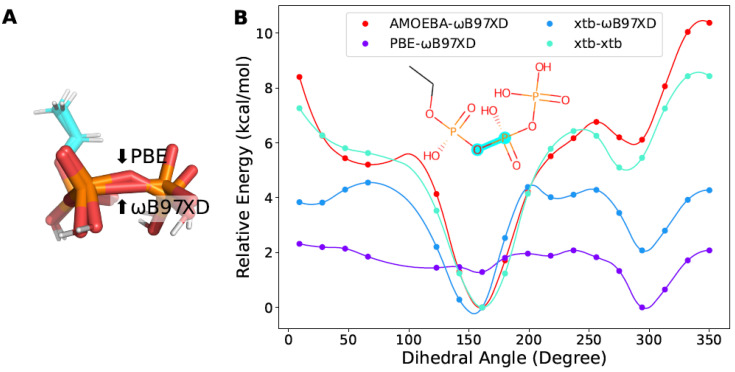
The torsion profile of the triphosphate fragment. (**A**) The optimized structures at the level of PBE/6-311G or ωB97XD/6-311G. (**B**) The torsion profile of the triphosphate fragment obtained with different methods. The scanned torsion bonds are highlighted in cyan. The same notation as in Figure 4 is used in the legends.

We also found that fragments containing more than one phosphoric acid groups were non-trivial with respect to optimization. On one hand, the P-O-P bond in the triphosphoric acid was flattened during optimization at the level of ωB97XD/6-311G (Figure 7A). One the other hand, a non-negligible number of points failed to converge at that level of theory with Psi4. Finally, we managed to optimize with Gaussian09 at the level of PBE/6-311G to obtain geometry of reasonable quality. However, the torsion profiles obtained by xtb diverged from those from DFT (Figure 7B). This problem might arise from the strong interactions between the phosphoric acid groups, making it hard to reach the minima without a polarizable continuum model in vacuum.

## 3. Materials and Methods

### 3.1. Dataset

We compiled the dataset used in this work by emphasizing both drug-likeness and chemistry coverage. We started with ∼30 drug molecules randomly selected from the DrugBank database [40], which were cut into fragments with Poltype 2. The fragmenter in Poltype 2 ensures that the electron density of the fragment close to the innermost rotatable bond is similar to the electron density of the parent molecule for the same bond, which reduces the DFT computation cost without sacrificing the accuracy of torsion energy scans of that bond. These drug molecules provided us ∼100 unique fragments, of which each has one or more torsions that were scanned. We further expanded our dataset by the addition of ∼100 small organic molecules retrieved from PubChem [41] based on the torsion chemistry uncovered by the drug fragments. Our final dataset was composed of 233 fragments with 344 torsions, which covers 124 unique torsions defined by the elements of the four atoms of the torsion angle *a-b-c-d*. The dataset can be found in Table S1.

### 3.2. Computational Details

In this work, we focused on the comparison between DFT, semi-empirical, and NNP methods. To this end, we chose a representative method for each of the three categories, namely, GFN2-xTB, ωB97XD, and ANI-2x. GFN2-xTB was published in 2019 as a variant to the tight-binding DFTB3 [42,43] scheme, which includes anisotropic second-order density fluctuation effects via short-range damped interactions of cumulative atomic multipole moments. GFN2-xTB was designed to account for properties around the energetic minimum, e.g., geometries. There have been several studies [36,44] demonstrating that the theoretically sophisticated GFN2-xTB performs better than or comparable with other semi-empirical methods such as AM1 [45], PM3 [46], DFTB3, etc. The ωB97XD functional showed overall better performance than most of the 200 density functionals, including PBE0-D3(BJ) [47,48], PBE-D3(BJ) [48,49], and M06-2X [50], according to the benchmark study published by Head-Gordon and coworkers in 2017 [38]. There have been other publications benchmarking [51] or discussing [52] the performance of ωB97XD. Overall, ωB97XD was considered to offer a good balance between computational cost and accuracy. ANI-2x is the most current version of ANI potentials, with the best performance and broadest chemistry coverage (including seven elements: C, H, O, N, S, F, and Cl).

The overall workflow is as follows. (i) Minimize the input structure at the MP2 [53]/6-31G* [54,55,56,57,58,59] level of theory to obtain a good initial geometry for the subsequent conformational energy scan. (ii) With all the other torsion dihedrals fixed, rotate the scanned torsion bond to generate structures along the dihedral angle at uniform intervals. The increment of the degree is dependent on the number of cosine terms used in torsion fitting (i.e., three terms in this work) and the number of other torsions around the scanned torsion, due to needing more points sampled than number of parameters being fit for AMOEBA. (iii) Minimize the structures with constraints on all torsions using AMOEBA, and then use the indicated optimization methods, i.e., xtb, ANI, or ωB97XD/6-311G [60,61,62,63]. The force constants for xtb were set as 5 kcal/mol/deg${}^{2}$. The convergence criteria for Psi4 are default, at {Delta E: $1.00\times 10^{-4}$ au, MAX Force: $2.50\times 10^{-3}$ au, RMS Force: $1.70\times 10^{-3}$ au, MAX Disp: $1.00\times 10^{-2}$ au, RMS Disp: $6.70\times 10^{-3}$ au}. The convergence criteria for xtb are default, at “normal”, {Econv: $5\times 10^{-6}$ au, Gconv: $1\times 10^{-3}$ au/α }. (iv) Calculate the single-point energies with the indicated methods, including xtb, ANI, and ωB97XD/6-311+G* [55,63,64,65,66].

GFN2-xTB was readily exploited with xtb software. All DFT calculations involved in this work were performed with Psi4 [67], except for those explicitly mentioned as using Gaussian09 [68]. ANI-2x was accessed by the TorchANI [69] Python module. For simplicity, xtb and ANI will be used to refer to GFN2-xTB and ANI-2x through the article, respectively.

### 3.3. Metrics

We mainly used RMSE and absolute error (AE) to analyze the results obtained from different methods. In typical torsion parameterization scenarios, the Boltzmann-weighted error is widely adopted as well, which emphasizes the energy deviation close to the equilibrium structures, since this conformational space is of great significance in equilibrium MD simulations. The AE and RMSE are given by

(1) $$AE\left(i\right)=|E_{i}-E_{i}^{DFT}|$$

(2) $$RMSE=\sqrt{\sum_{i=0}^{N}\frac{1}{N}AE{\left(i\right)}^{2}}$$

The Boltzmann-weighted versions, denoted as a superscript of B, are given by

(3) $$AE^{B}\left(i\right)=\frac{N\exp(-\frac{E_{i}^{DFT}}{k_{B}T})}{\sum_{i=0}^{N}\exp\left(-\frac{E_{i}^{DFT}}{k_{B}T}\right)}\left|E_{i}-E_{i}^{DFT}\right|$$

(4) $$RMSE^{B}=\sqrt{\sum_{i=0}^{N}\frac{\exp(-\frac{E_{i}^{DFT}}{k_{B}T})}{\sum_{i=0}^{N}\exp\left(-\frac{E_{i}^{DFT}}{k_{B}T}\right)}AE{\left(i\right)}^{2}}$$

## 4. Conclusions

Based on what was reported in this work, we would like to recommend the sequential protocol of optimization with GFN2-xTB and single-point energy at ωB97XD/6-311+G* as a promising alternative for large-scale conformational scans. With running time reduced by factors of hundreds, this protocol can provide geometry in excellent agreement with that obtained by ωB97XD/6-311G, and accurate DFT single-point energy. Other combinations of comparable semi-empirical methods and DFT methods may be able to provide similar results, but relevant benchmark studies are necessary to draw a general conclusion. It should also be noted that researchers need pay attention to the chemistry of their dataset, since certain functional groups can be tricky for all methods. We hope that this work can benefit researchers in need of large-scale conformational energy scans.

## Data Availability

All data not reported can be requested from the corresponding author.

## Supplementary Materials

The following supporting information can be downloaded at: https://www.mdpi.com/article/10.3390/molecules27238567/s1, Figure S1: The distribution of the elapsed times of optimization with ANI-2x and ASE Python module; Table S1: The SMILES of the fragments composing the dataset in this work.
