# Supplementary material for: An Efficient Approach to Large-Scale Ab Initio Conformational Energy Profiles of Small Molecules
Source: Molecules. 2022 Dec 5;27(23):8567. doi: 10.3390/molecules27238567 (PMC9738817; doi:10.3390/molecules27238567)
Supplement: Supplementary file 1 [file molecules-27-08567-s001.zip › molecules-2030030-supplementary.pdf]

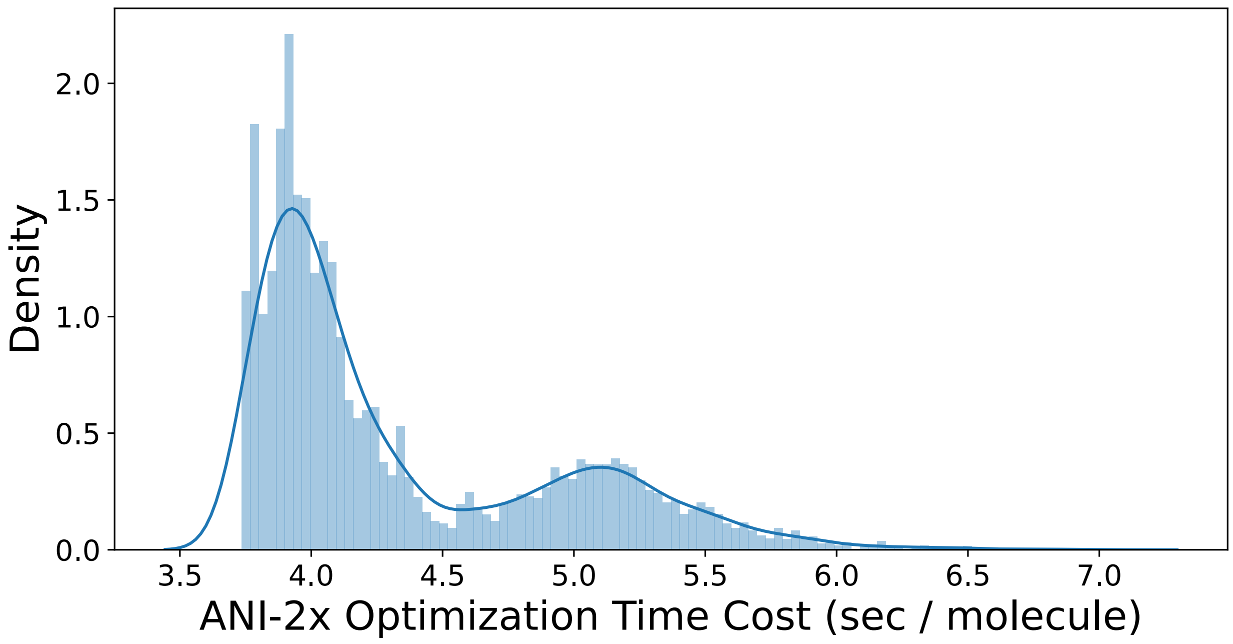

Figure S1. The distribution of the elapsed times of optimization with ANI-2x and ASE Python module. Since ANI-2x does not provide an official timer, the time was determined by the time elapsed from entering the command to the full exit of the program, which might overestimate the time cost.

Table S1. The SMILES of the fragments composing the dataset in this work. The scanned torsion bonds are indicated by the atoms with an atom map number.

**Index**	**SMILES**
1	O=[C:3](c1ccccc1)[c:2]1ccccc1
2	O=[C:3](O)[c:2]1ccccc1O
3	Nc1cccc[c:2]1[CH2:3]C(=O)O
4	F[C:3](F)(F)[c:2]1ccccc1
5	c1cc[c:2]([CH3:3])cc1
6	O=Cc1cccc(O)[c:2]1[CH3:3]
7	COc1cccc[c:5]1[OH:10]
8	c1cc[c:2]([OH:3])cc1
9	Cc1cc[c:2]([OH:8])cc1
10	O=[S:2](=O)(Nc1ccccn1)[c:1]1ccccc1
11	O=Cc1con[c:2]1-[c:3]1c(F)cccc1Cl
12	O=Cc1cno[c:2]1[CH3:3]
13	c1nn[c:2]([CH3:3])s1
14	C/C=[C:2](/c1ccccn1)[c:3]1ccccc1
15	C/C=[C:2](\c1ccccc1)[c:5]1ccccn1

16	CC/C(C=O)=[C:2](\[C@](O)(C=O)CC)[OH:3]
17	C=[CH:2][CH2:3]I
18	C=[CH:2][CH2:3]SS
19	CN/[C:2](=C(\C)C=O)[CH3:3]
20	CC(C)(O)[C:2](=O)[CH2:3]Cl
21	C[C:2](=O)[CH3:3]
22	C[C:7](=O)[N:9](C)C
23	C[C:1](=O)[NH2:3]
24	CC[C:1](=O)[NH2:3]
25	C[C@@H](c1ccccc1)[C:5](=O)[OH:16]
26	C[C:2](=O)[O:4]SCF
27	C[C:2](=O)[S:4]C
28	C[C:2](=S)[O:4]C
29	C[N:4](C)[CH:2]=O
30	O=[CH:1][NH2:4]
31	O=[CH:1][SH:3]
32	S=[CH:1][SH:3]
33	CN[C:2](=O)[c:3]1c(-c2ccccc2)noc1C
34	C[CH2:5][C:1](N)=O
35	N[C:1](=O)[CH3:5]
36	CN(C)[C:7](=O)[CH3:14]
37	CN[C:3](=O)[CH3:5]
38	CCN(CC)[C:3](=O)[N:6](C)C
39	CN[C:1](=O)[N:2](C)N=O
40	O=c1cc[nH]c[c:5]1[C:3](=O)O
41	CO[C:2](=O)[c:3]1cccnc1
42	O=[C:3](O)[CH2:5]c1ccccc1
43	C[C@@H:2](Br)[CH2:3]C
44	CC[C@H:2](Br)[CH3:4]
45	O=[CH:2][CH2:1]Br
46	Br[CH2:1][CH2:2]Br
47	Br[CH2:1][CH2:2]I
48	CC[C@@:2]1([CH3:3])[C@H](C)CC[C@]1(O)C=O
49	C=C[C@@:2](C(=C)C)(C(C)(C)Cl)[CH3:3]
50	C[C:1](C)(C)[CH3:4]
51	CC(C)[C@@:2](C)(c1ccccc1)[OH:6]
52	C=C(O)[C@:2](C(C)=O)(C(C)C)[OH:3]
53	CC(=O)[C@@:2]1([OH:3])[C@H](C)CCC1(C)C
54	C[C@H:2](C(=O)O)[c:3]1ccccc1

55	C=C(C=O)[CH:2](C(=C)C=O)[c:3]1cccc2nonc21
56	O=C1C=CC(=O)[CH:2]1[c:3]1ccccc1
57	C[C@@H:2](c1ccccc1)[C:5](=O)O
58	O=C[C@]1(O)CCC[C@H:2]1[CH3:3]
59	C[CH:1](C)[CH3:3]
60	C=C(O)[C@H:2](C(C)C)[N:3](C)C
61	CC(C)[CH:2](C(C)C)[OH:3]
62	CC[C@@H:2](C(C)(C)Cl)[OH:3]
63	O=C1NC(=O)[C:2](c2ccccc2)([c:3]2ccccc2)N1
64	C[C:2](C)(NC=O)[CH3:3]
65	CO[C:2](C)(c1ccccc1)[c:3]1ccccc1
66	CO[C:2](c1ccccc1)(c1ccccc1)[CH3:6]
67	CC(C)[C@:2](O)(c1ccccc1)[CH3:3]
68	C[C:2](C)(O)[CH3:5]
69	C[C:2]1([CH3:3])SCN[C@H]1C=O
70	c1cc[c:3]([CH2:2]c2ccccn2)cc1
71	CC[CH2:2][c:3]1ccccc1
72	NC[CH2:2][c:3]1ccccc1
73	C[CH2:1][c:2]1ccccc1
74	c1ccc([CH2:2][c:3]2nccc3ccccc32)cc1
75	CC[C:4](=O)[CH2:3]C
76	C[CH2:2][CH:4]=O
77	C[CH2:3][C:5](=O)O
78	CC[CH2:3][CH2:2]C[NH3+]
79	CC[CH2:2][CH2:3]CC
80	C[C@@H](N)[CH2:2][CH2:3]c1ccccc1
81	C[CH2:1][CH2:2]C
82	C[CH2:3][CH2:2]CO
83	CC[CH2:1][CH2:3]C
84	C[CH2:2][CH2:3]Cl
85	NC[CH2:2][CH3:3]
86	C[C@@H](Br)[CH2:3][CH3:5]
87	CC[CH2:2][CH3:4]
88	OCC[CH2:3][CH3:5]
89	c1ccc([CH2:1][CH3:5])cc1
90	O=C[CH2:2][CH3:3]
91	CCC(=O)[CH2:3][CH3:6]
92	NC(=O)[CH2:5][CH3:6]
93	O=C(O)[CH2:3][CH3:4]

94	CCC[CH2:3][CH3:5]
95	C[CH2:1][CH3:2]
96	OC[CH2:2][CH3:4]
97	C[CH2:2][CH2:3]N
98	CC[CH2:2][CH2:4]O
99	C[CH2:2][CH2:3]O
100	NC[CH2:2][NH2:3]
101	BrC[CH2:1][NH2:4]
102	FC[CH2:1][NH2:4]
103	C[CH2:1][O:3]C
104	ClC[CH2:1][OH:4]
105	CP(=O)(O)[O:3][CH2:2]C
106	CS(=O)(=O)[O:3][CH2:2]C
107	N#C[S:3][CH2:2]CF
108	SC[CH2:1][SH:4]
109	BrC[CH2:1][SH:4]
110	C[CH2:2][SH:3]
111	O=C[CH2:1][SH:4]
112	ClC[CH2:1][SH:4]
113	C=C[CH2:3][S:4]S
114	CC1(C)SCN(C=O)[C@H:2]1[C:3](=O)O
115	CN1CCC[C@@H:2]1[CH2:3]CO
116	CN[C@H:2](C)[CH2:3]Cc1ccccc1
117	CN[C@@H:2](C)[C@H:3](O)c1ccccc1
118	CC[C@H:2](NC)[CH3:3]
119	CN[C@H:2]([C@H](O)c1ccccc1)[CH3:5]
120	NC[C@@H:3](O)[c:6]1ccccc1
121	C[CH:2](OC=O)[CH3:3]
122	C[CH:2](O)[CH3:3]
123	CO[C@@H:2]([C@H](C)O)[CH2:3]O
124	I[CH2:4][CH:2]1CS1
125	Cl[CH2:1][CH2:2]Br
126	Cl[CH2:1][CH2:2]Cl
127	Cl[CH2:1][CH2:2]I
128	O=[CH:2][CH2:1]F
129	F[CH2:1][CH2:2]Br
130	F[CH2:1][CH2:2]Cl
131	F[CH2:1][CH2:2]F
132	F[CH2:2][CH3:3]

133	F[CH2:1][CH2:2]I
134	N#CS[CH2:2][CH2:1]F
135	O=[S:3](=O)(OS)[CH2:1]F
136	Oc1cc[c:7]([CH3:1])cc1
137	O=[CH:3][CH3:2]
138	O=[C:4](O)[CH3:3]
139	O=[C:2](OSCF)[CH3:1]
140	CO[C:2](=S)[CH3:1]
141	CS[C:2](=O)[CH3:1]
142	O=C(O)[C@H:2](c1ccccc1)[CH3:1]
143	ClC[CH2:2][CH3:1]
144	Cl[CH2:2][CH3:1]
145	[CH3:1][CH3:2]
146	I[CH2:2][CH3:1]
147	CP(=O)(O)O[CH2:2][CH3:1]
148	CS(=O)(=O)O[CH2:2][CH3:1]
149	S[CH2:2][CH3:1]
150	O=[N:2][CH3:1]
151	CN[NH:2][CH3:1]
152	N[NH:2][CH3:1]
153	SOS[NH:2][CH3:1]
154	POC[O:2][CH3:1]
155	NS(=O)(=O)[O:2][CH3:1]
156	COS[O:2][CH3:1]
157	CS(=O)[O:2][CH3:1]
158	CS(=O)(=O)[O:2][CH3:1]
159	SC[S:2][CH3:1]
160	C[S:2](=O)[CH3:1]
161	O[S:2][CH3:1]
162	O=[P+]([O-])O[S:2][CH3:1]
163	CSO[S:2][CH3:1]
164	S[S:2][CH3:1]
165	O=[CH:2][CH2:1]I
166	I[CH2:1][CH2:2]I
167	CN(C)[CH2:2]/[CH:3]=C(\c1ccccc1)c1ccccn1
168	C=[CH:3][CH2:2]N(C)C
169	N[CH2:1][CH2:2]Br
170	C[CH2:3][CH2:2]NC=O
171	CC[CH2:3][CH2:2][N+](C)(C)C

172	CN(C)[CH2:2][CH2:3]c1ccccc1
173	CN[CH2:2][CH2:3]c1ccccc1
174	C[C@H](N)[C@H:3](O)[CH2:2]N(C)C
175	CN[CH2:2][C@@H:3](O)c1ccccc1
176	N[CH2:1][CH2:2]F
177	CN(C=O)[CH2:2][CH3:3]
178	N[CH2:2][CH3:3]
179	N[CH2:1][CH2:2]N
180	CN(C)[CH2:2][CH2:3]Oc1ccccc1
181	O[CH2:1][CH2:2]Cl
182	CCO[CH2:3][CH3:5]
183	O[CH2:2][CH3:3]
184	CO[CH2:1][CH3:2]
185	CO[CH2:3][O:4]P
186	O[CH2:1][SH:3]
187	O=[CH:2][CH2:1]S
188	S[CH2:1][CH2:2]Br
189	S[CH2:1][CH2:2]Cl
190	S[CH2:1][CH2:2]S
191	S[CH2:1][OH:2]
192	CS[CH2:3][SH:4]
193	O=c1cc[n:2]([CH:3]2CC2)c2ccccc12
194	c1ccc2c(c1)cc[n:2]2[CH3:3]
195	Sc1cnc[n:2]1[CH3:3]
196	CC[N:2](CC)[c:3]1cc2[nH]ccc(=O)c2cc1F
197	CC[N:2](CC)[c:3]1ccccc1
198	CC[N:2](CC)/[C:3](=N/c1ccccc1)c1ccccc1
199	C[N:2](C)[CH:4]=S
200	CC[N:2](CC)[C:3](=O)N(C)C
201	CC(C)[CH2:3][N:2](c1ccccc1)c1ccccc1
202	CC(=O)[N:9](C)[CH3:10]
203	C[N:4](C=O)[CH3:9]
204	C[N:2](C=S)[CH3:3]
205	CNC(=O)[N:2](C)[N:3]=O
206	Cc1cccc[c:3]1[NH:2]c1ccccc1
207	Clc1cccc(Cl)[c:3]1[NH:2]c1ccccc1
208	CC(C)[NH:2][C:3](=O)c1ccccc1
209	C[C@@H](C=O)[NH:2][C:3](=O)c1cnoc1
210	C[NH:2][C:3](C)=O

211	C[NH:2][CH:4]=O
212	CC[NH:2][C:3](=O)N[SH](=O)=O
213	C[C@H](O)[C@H](C(O)O)[NH:2][C:3](=O)N(C)N=O
214	CC(=O)[NH:2][C:3](C)(C)C
215	C[C@@H](O)[C@H:3](CS)[NH:2]C(=O)c1ccccc1
216	O=C(c1cnoc1)[NH:2][C@@H:3]1C(=O)N[C@@H]1S
217	CC[CH2:3][NH:2]C(N)=O
218	CC(=O)[NH:2][CH3:4]
219	O=C[NH:2][CH3:3]
220	NC(=O)[N:2](N=O)[CH3:3]
221	c1cc[c:3]([NH2:2])cc1
222	O=CCc1cccc(C(=O)c2ccccc2)[c:3]1[NH2:2]
223	CS(=O)(=O)[NH:3][c:7]1ccccc1
224	O=S(=O)(c1ccccc1)[NH:3][c:7]1ccccn1
225	CN[C:3](=O)[NH:2]S(=O)(=O)c1ccccc1
226	c1ccc(/N=[N:2]/[c:3]2ccccc2)cc1
227	O=[N+:2]([O-])[c:3]1nc[nH]c1S
228	C=C[CH2:3][N:2]1CCCC1
229	CC[N:2](C)[CH2:3]Cc1ccccc1
230	CC[N:2](C)[CH2:3]CO
231	CC[N:2]([C@@H](C)C=O)[CH2:3][C@H](C)O
232	C=C[CH2:3][N:2](CC)C(C)C
233	CC[N:2](CC)[CH3:3]
234	C[C@H]1CCC[N:2]1[CH3:3]
235	CC(C)[N:2](C)[CH3:3]
236	CC[N:2](C(C)C)[CH3:3]
237	C[N:1](C)[CH3:3]
238	CC[CH2:3][N+:2](C)(C)C
239	CC[N+:2](C)(C)[CH3:3]
240	CC[NH:2][C@H:3](C)CC
241	CC[NH:2][C@@H:3](C)[C@H](O)c1ccccc1
242	CC(C)[NH:2][CH2:3][C@@H](O)c1ccccc1
243	CC(C)[NH:2][CH2:3]Cc1ccccc1
244	C[NH:1][CH3:2]
245	C[NH:2][NH:3]C
246	C[NH:2][NH2:3]
247	C[NH:2][S:3]OS
248	C[CH2:2][NH2:1]
249	CC[CH2:3][NH2:1]

250	O=P(O)(O)O[S:2][NH2:1]
251	O=[S:2](O)[NH2:1]
252	C[O:2][c:3]1cc2cnccc2cc1O
253	C[O:2][c:3]1cc2ccncc2cc1O
254	C[O:2][c:3]1ccccc1O
255	CC[O:2][c:3]1ccccc1
256	C=C(C)[C:3](=O)[O:2]C(C)C
257	C=C(C)[C:3](=O)[O:2]C
258	CC[O:2][C:3](=O)c1cccnc1
259	CC(=O)[O:2][CH:3](C)C
260	CC(C)[CH2:3][O:2]C(=O)c1cccnc1
261	c1ccc([O:2][CH3:3])cc1
262	CC(=O)[O:2][CH3:3]
263	CC(=S)[O:4][CH3:5]
264	CC(=O)[O:4][S:5]CF
265	O=Cc1cccc[c:3]1[OH:2]
266	Cc1cccc[c:3]1[OH:2]
267	O=c1cc[nH]cc1[C:3](=O)[OH:2]
268	O=[C:3](c1ccccc1)[OH:2]
269	C[C@@H](N)[C:3](=O)[OH:2]
270	O=[C:3](Cc1ccccc1)[OH:2]
271	C[C:4](=O)[OH:1]
272	CC[C:5](=O)[OH:1]
273	O=[CH:3][OH:1]
274	CC[O:2][C:3](C)(c1ccccc1)c1ccccc1
275	CC[C@:3]1([O:2]C)c2cccc3[nH]cc(c23)C[C@H]1N
276	CC[CH2:3][O:2]C(C)(c1ccccc1)c1ccccc1
277	CC[O:1][CH2:3]C
278	CC(C)(C)[O:2][CH3:3]
279	CC[O:3][CH3:4]
280	C[O:1][CH3:2]
281	C[O:2][CH2:3]OP
282	CC[O:3][P:4](C)(=O)O
283	COC[O:4][PH2:5]
284	C[O:2][S:3](C)=O
285	C[O:2][S:3](C)(=O)=O
286	CC[O:3][S:4](C)(=O)=O
287	C[O:2][S:3](N)(=O)=O
288	CO[S:3][O:2]C

289	C[C:2](C)(C)[OH:1]
290	C[C@H](N)[C@H:3]([C@@H](C)O)[OH:2]
291	C[C@@H](O)[CH:3]([C@@H](C)O)[OH:2]
292	NC[C@H:3](c1ccccc1)[OH:2]
293	C[CH:2](C)[OH:1]
294	C[C@H](O)[CH2:3][OH:2]
295	CCC[CH2:4][OH:1]
296	C[CH2:2][OH:1]
297	CC[CH2:3][OH:1]
298	CO[C@@H:3]([C@@H](C)N)[OH:2]
299	[OH:1][CH3:2]
300	O=[P:2][OH:1]
301	O=[S:2](=O)(OP)[OH:1]
302	COP(O)[O:5][CH3:6]
303	O=P[O:3][SH:4]
304	CS[O:3][P+:4](=O)[O-]
305	O=S(=O)(O)[O:5][PH2:6]
306	NS[O:3][P:4](=O)(O)O
307	O=S(=O)(CF)[O:6][SH:7]
308	CNS[O:4][SH:5]
309	O=[P:2][O:3]S
310	CCO[P:4](C)(=O)[OH:7]
311	CO[P:3](O)[O:5]C
312	CO[P:3](OC)[OH:4]
313	CCO[P:4](=O)(O)[CH3:6]
314	NSO[P:4](=O)(O)[OH:6]
315	CC[S:2][c:3]1ccccc1
316	Cn1cnc([NH+]=O)[c:3]1[S:2]c1ccncn1
317	c1nc2nc[nH]c2[c:3]([S:2]c2cnc[nH]2)n1
318	C[C@@H](N)[CH2:3][S:2]c1ccccc1
319	C[S:1][CH3:2]
320	CC(=O)[S:4][CH3:5]
321	C[S:2][CH2:3]S
322	C[S:2][OH:3]
323	C[S:2][O:3][P+](=O)[O-]
324	CS[O:3][S:2]C
325	C[S:2][S:3]C
326	C[S:2][SH:3]
327	C=CC[S:4][SH:5]

328	O=[S:2](=O)(c1ccccc1)[NH:3]c1nncs1
329	O=[S:2](=O)(c1ccccc1)[NH:3]c1ccccn1
330	C[S:2](=O)(=O)[NH:3]c1ccccc1
331	O=[S:3](=O)(CF)[O:6]S
332	[SH:1][CH3:2]
333	N[S:2][O:3]P(=O)(O)O
334	CN[S:3][O:4]S
335	N[S:2](=O)[OH:4]
336	O=[S:2](=O)(Nc1nncs1)[c:6]1ccccc1
337	O=[S:2](=O)(Nc1ccccc1)[CH3:6]
338	CC(=O)O[S:5][CH2:6]F
339	CO[S:3](=O)[CH3:5]
340	O=[S:2](O)[OH:4]
341	CO[S:3](=O)(=O)[CH3:6]
342	CCO[S:4](=O)(=O)[CH3:7]
343	CO[S:3](=O)(=O)[NH2:6]
344	O=[S:2](=O)(O)[O:5]P
